# Adverse Childhood Experiences, DNA Methylation, and Depressive Symptoms in Black Pregnant Women

**DOI:** 10.3390/epigenomes9040048

**Published:** 2025-11-27

**Authors:** Alexandra L. Nowak, Marvin A. Schilt-Solberg, Xiaoyu Liang, Fabiola Magaña, Dawn P. Misra, Carmen Giurgescu

**Affiliations:** 1 Department of Family and Community Health Nursing, Marcella Niehoff School of Nursing, Loyola University, Chicago, IL 60153, USA; fmagana1@luc.edu; 2 Department of Systems, Population, and Leadership, School of Nursing, University of Michigan, Ann Arbor, MI 48109, USA; solberma@umich.edu; 3 Department of Epidemiology and Biostatistics, College of Human Medicine, Michigan State University, East Lansing, MI 48824, USA; liangx20@msu.edu (X.L.); misradaw@msu.edu (D.P.M.); 4 College of Nursing, University of Central Florida, Orlando, FL 32816, USA; carmen.giurgescu@ucf.edu

**Keywords:** adverse childhood experiences, ACEs, childhood maltreatment, prenatal depression, pregnancy, DNA methylation, epigenetics, African American, women

## Abstract

Background: Prenatal depression, affecting up to a quarter of all pregnancies in the United States, contributes to morbidity and mortality and is associated with increased risk of adverse birth and long-term mental health outcomes. Adverse childhood experiences (ACEs, or experiences of abuse, neglect, or family dysfunction experienced prior to age 18) are a strong predictor of adult depression and adverse health outcomes. The present study investigated whether epigenetic modification in the form of DNA methylation (DNAm) of four stress-related, glucocorticoid pathway genes (*CRH*, *CRHR1*, *FKBP5*, *NR3C1*) mediates associations between ACEs and depressive symptoms among Black pregnant women. Methods: Using a cross-sectional design, we examined the mediating role of DNAm on the relationship between depressive symptoms (Center for Epidemiologic Studies Depression Scale (CES-D)) and ACEs (Centers for Disease Control and Prevention 10-item questionnaire), in a subsample (*n* = 61) of Black pregnant women who were participants of the Biosocial Impacts of Black Births (BIBB) study. Results: A significant association was found between ACEs and depressive symptoms scores (TE α_X = 2.29 with p_TE = 6.60 × 10^5^). DNAm on five CpG sites within two genes significantly mediated the relationship between ACEs and depressive symptoms (cg03238273 on *CRHR1*, and cg08845721, cg16594263, cg19820298, and cg23430507 on *NR3C1*). Conclusions: This study provides evidence that DNAm partially mediated the association of ACEs and depressive symptoms during pregnancy among Black pregnant women. Understanding the molecular pathways underlying the mediating effect of ACEs on depressive symptoms among Black pregnant women can illuminate biological markers that help identify and treat pregnant women who are at an increased risk for depression following childhood trauma.

## 1. Introduction

Prenatal depression (PD), depression occurring during pregnancy, contributes to maternal morbidity and mortality and is associated with increased risk of adverse birth outcomes (e.g., preterm birth), diminished fetal and neonatal attachment, and long-term mental health problems in offspring [1,2,3,4]. PD rates in the United States are estimated at 10–25% of pregnant women, with higher rates for Black women compared to non-Hispanic White women [5]. A large study (*n* = 116,449) leveraging electronic health records for Kaiser Permanente Northern California abstracted diagnoses of depression, anxiety, and comorbid depression/anxiety before, during, and after pregnancy. Non-Hispanic Black pregnant women had a higher risk of PD (RR = 1.35, 95% CI: 1.26–1.44) and severe PD (RR = 159, 95% CI: 145–175) compared to non-Hispanic White women [6]. One possible mechanism explaining higher rates of PD in women is the high prevalence of adverse childhood experiences (ACEs).

### 1.1. Adverse Childhood Experiences

ACEs are stressors in the form of physical, verbal, or sexual abuse, neglect, or family dysfunction occurring prior to age 18. ACEs may be more prevalent for Black women. In the 23 states participating in the 2011–2014 U.S. Behavioral Risk Factor Surveillance Survey, Black women reported higher rates of ACEs compared to White women [7]. ACEs have long been known to contribute to adult depression [8,9,10,11,12], including prenatal and postpartum depression [13,14]. Indeed, in a recent study of 294 women (59% White; 16% Black), researchers found that those with high ACE scores were more likely to experience antenatal depression than those with low ACE scores (10.3% vs. 4.3%, *p* = 0.008) [15]. Another study of 199 racially diverse pregnant women (67.8% Black) found that ACEs among Black women (but not White women) were associated with PD (β = 3.85, *p =* 0.009) [16]. Evidence supports the association of ACEs and PD. However, the underlying biological mechanisms are not known. The Hypothalamic–Pituitary–Adrenal (HPA) axis stress response system is a plausible pathway by which ACEs affect PD.

### 1.2. Toxic Stress

The HPA axis is a neuroendocrine regulatory system that, together with its negative feedback mechanism, works to maintain a homeostatic stress response [17]. Briefly, Corticotropin-Releasing Hormone (CRH) is expressed in response to physical and psychological stressors [18] and binds with its receptors. CRH has a high affinity for its CRHR1 receptor, and CRHR1 is the main CRH receptor in the brain [19]. CRH-CRHR1 binding activates the release of systemic adrenocorticotropic hormone (ACTH), which in turn binds to receptors in the adrenal cortex and stimulates the release of glucocorticoids (GCs; cortisol) [20]. Systemic cortisol then binds with Nuclear Receptor Subfamily 3 Group C Member 1 (*NR3C1*), commonly known as the glucocorticoid receptor (GR), forming a complex with FKBP5 (FK06 Prolyl Isomerase 5), triggering GR hyperphosphorylation and translocation into the nucleus [21]. GR binding with glucocorticoid response elements and transcription factors, repressing transcription of *CRH* and counteracting the activity of the HPA axis once the stressful event has passed [22]. However, under prolonged exposure to stressors, the GR develops resistance to cortisol, reducing the number of GR-GC binding opportunities. Decreased numbers of GR-bound ligands reduce transcriptional repression of CRH, further reducing the negative feedback response [23]. Prolonged exposures to stressors result in the inability of this negative feedback mechanism to compensate, leading to what has been termed “allostatic load” or “toxic stress” and subsequent dysregulation of the brain and other organ systems [24,25,26]. The HPA axis responds to early life stress and the translation of early stress into long-term mental health outcomes, including PD [27,28,29]. The HPA axis regulatory response is a likely pathway by which toxic stress leads to long-term mental health outcomes, but the biological mechanism underlying the association of ACEs and PD is unknown. Epigenetic modifications of the HPA axis, stress-related genes, may underlie this association.

### 1.3. DNA Methylation

Epigenetics refers to modifications to DNA that regulate gene expression without altering the gene sequence [30]. Environmental exposures (e.g., infection, substance use, and psychosocial stress) promulgate epigenetic alterations [31]. Importantly, epigenetic modifications are potentially reversible [32]. DNA methylation (DNAm) is an epigenetic mechanism wherein a methyl molecule forms or breaks a covalent bond with the fifth carbon atom of cytosine bases that precede guanine (CpG) nucleotide sites [30]. CpG sites occur commonly in CpG islands (regions rich in CpG dinucleotides). DNAm at multiple CpG sites at or near CpG islands may result in repressed or silenced transcription of the gene and its resulting protein [30].

Research suggests that DNAm plays an important role in the long-term effects of ACEs [33,34,35,36]. Studies have documented DNAm differences associated with ACEs in stress-related genes involved in glucocorticoid signaling [34]. Pioneering research demonstrated altered methylation of the glucocorticoid receptor (*NR3C1*) in adults with ACEs [37]. Parade et al. (2016) found associations between *NR3C1* methylation and behavioral issues in children with ACEs [38]. Parent et al. (2017) found a link between ACEs and changes in *NR3C1* methylation over time [39]. Subsequent systematic reviews and meta-analyses have confirmed these findings [33,40,41,42,43,44]. More recent research has investigated a wider range of genes and their effects associated with ACEs, including the *NR3C1* co-chaperone FK506 binding protein 5 (*FKBP5*) [38,45,46,47], Corticotropin-Releasing Hormone (*CRH*) [34], and its receptor Corticotropin-Releasing Hormone Receptor 1 (*CRHR1*) [46].

Genes involved in the operation of the HPA axis have long been associated with depression [48]. Depression is a type of stress that both increases HPA axis activity and cortisol and is associated with preterm birth (less than 37 completed weeks gestation) [49,50,51,52]. DNAm has been identified as a biological mechanism that may underlie the associations between PD and preterm birth via increased cortisol output or an increase in placental CRH contributing to preterm labor [51,53,54]. Several studies have suggested that maternal depression is associated with DNAm of *NR3C1*. For example, Oberlander et al. (2008) reported increased methylation at the *NR3C1* promoter region in human cord blood of women reporting depressive symptoms [55]. Prenatal depression was positively correlated with the degree of methylation in maternal blood, and depression predicted methylation in the *NR3C1* promoter region in Chinese women during the COVID-19 lockdown [56].

Interestingly, Castro et al. (2024) examined the maternal blood of 161 women in late pregnancy and found that depressive symptoms during pregnancy were associated with increased DNAm and found an interaction between current distress levels due to each traumatic childhood event and DNAm within the *NR3C1* promoter region [57]. In other HPA axis genes, *CRHR1* has been associated with depression, particularly after childhood trauma [58,59]. HPA axis genes are associated with maternal ACEs and depression [34,57,58]. However, no studies have examined DNAm as a potential mediator of ACEs and PD. The purpose of this study was to examine whether DNAm of four stress-related, glucocorticoid pathway genes (*CRH*, *CRHR1*, *FKBP5*, *NR3C1*) mediates any associations between ACEs and depressive symptoms among Black pregnant women.

## 2. Results

**Figure 1:**
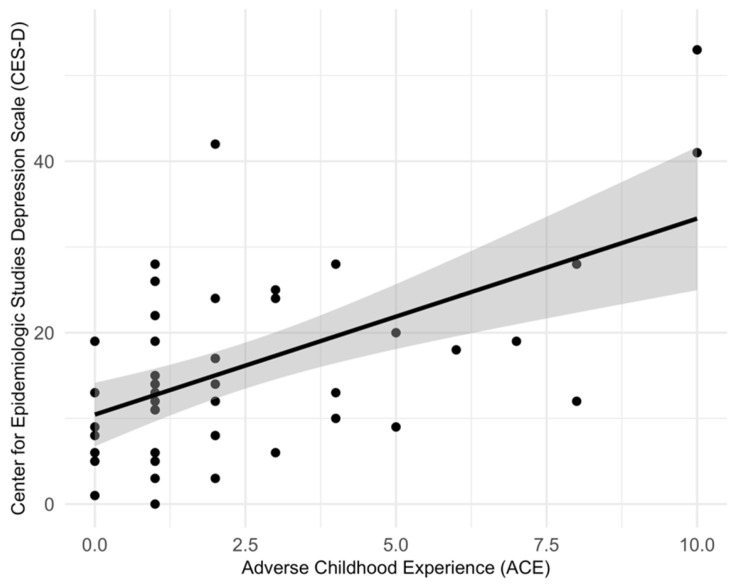
Scatter plot of adverse childhood experiences (ACE) and Center for Epidemiologic Studies Depression Scale (CES-D) scores among Black pregnant women. The solid line shows the fitted linear regression line and the grey band shows the 95 percent confidence interval. Pearson correlation coefficient *r* = 0.55, *p* = 6.60 × 10^5^.

The mean age of participants was 27.8 years. The mean gestational age at data collection was 13.6 weeks. Most women reported that they were never married (59.3%). Others reported being married or living with their partner (34%) or divorced or separated (4%). Most women reported that they were currently working (52.4%). Fifty-two women (85.2%) had an annual household income of less than USD 30,000 and 21 (34.4%) of those reported an income of USD < 10,000. A total of 11 out of 49 women reported experiencing 4 or more ACEs (22.5%), and 30 reported 1–3 ACEs (61.2%). CES-D scores ranged from 0 to 55. Twenty-six women (44.1%) had CES-D scores greater than or equal to 16, and 17 women (28.8%) had CES-D scores of >23 (Table 1) (Figure 1).

**Figure 2:**
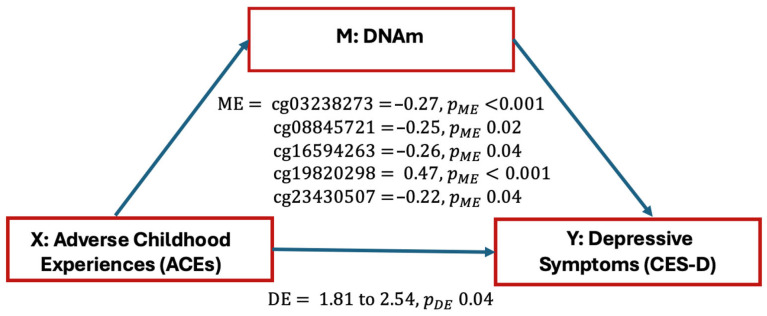
Mediation analysis results for the effect of adverse childhood experiences (Xs) on depressive symptoms (Y) through DNA methylation (M), showing both the direct effects (DEs) and mediation effects (MEs).

**Figure 3:**
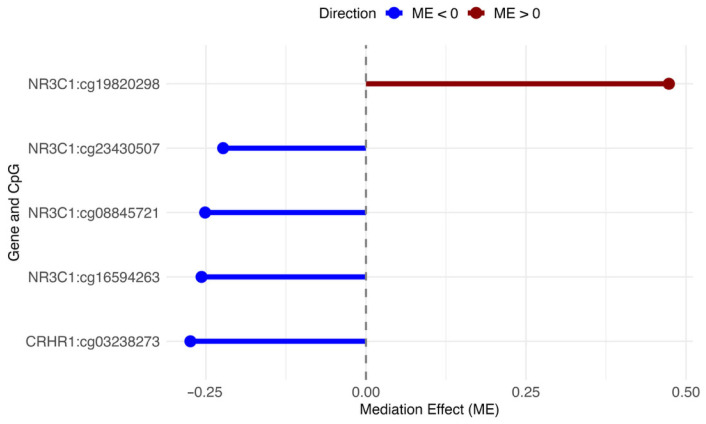
Mediation effects (ME) of CpG methylation on the link between Adverse. Childhood Experiences (ACEs) and the Center for Epidemiologic Studies Depression Scale (CES-D). Rows list CpGs as Gene: Probe. Dots show ME. Lines show size. The dashed line marks 0. Red = ME > 0. Blue = ME < 0.

We found a total of 330 CpG sites located in *FKBP5*, *NR3C1*, *CRH*, or *CRHR1* (GRCh37/hg19 assembly). We then estimated the effect of ACEs on depressive symptoms that was explained by the DNAm via mediation analysis. First, we tested the association between ACEs and CES-D scores in the *FKBP5*, *NR3C1*, *CRH*, and *CRHR1* genes and found a significant association between ACEs and CES-D scores (TE $\alpha_{X}=2.29$ with $p_{TE}=6.60$ × 10^5^). Of 330 CpG sites in the four genes, we found five CpG sites in two genes (*NR3C1*, *CRHR1*) to significantly mediate the relationship between ACEs and depressive symptoms–one CpG site (cg03238273) in the *CRHR1* gene and four CpG sites (cg08845721, cg16594263, cg19820298, cg23430507) in the *NR3C1* gene (Table 2). For cg03238273 (*CRHR1*), the ME was −0.27 with a highly significant *p*-value ($p_{ME}$ < 2 × 10^−16^), while the DE was 2.56 ($p_{DE}$ < 2 × 10^−16^), indicating that both mediated and direct pathways contribute to depressive symptoms. Similarly, multiple probes in the *NR3C1* gene showed significant mediation effects. For example, cg08845721 (ME = −0.25, $p_{ME}$ = 0.02) and cg19820298 (ME = 0.47, $p_{ME}$ < 2 × 10^−16^), along with strong direct effects (DE ranging from 1.81 to 2.54, with $p_{DE}$ values ≤ 0.04) (Figure 2 and Figure 3).

An exploratory epigenome-wide analysis examined associations of ACEs and DNAm. However, no CpG reached significance after correcting for multiple comparisons (FDR 5%/Bonferroni).

## 3. Discussion

This study indicates correlations among ACEs, depressive symptoms, and methylation status within two stress-related, glucocorticoid pathway genes (*NR3C1*, *CRHR1*) during pregnancy among Black women. We found that one CpG site, cg03238173, within the *CRHR1* gene and four CpG sites—cg08845721, cg16594263, cg19820298, cg23430507—within the *NR3C1* gene partially mediated the relationship of ACEs on depressive symptoms.

Epigenetic research on early life adversities has focused on the *NR3C1* gene and its effect on HPA axis regulation and the stress response [37,55,60,61]. Most associations with DNAm of *NR3C1* have focused on the proximal promoter region, which contains nine first exon alternative promoters, labeled A-J (excluding G), that enable transcription in a tissue-specific fashion [30]. Seven of the first exons are within a CpG island that is located upstream of the *NR3C1* start codon [30]. The 1F exon has been linked to early life stress and depression [43,62]. For example, McGowan et al. (2009) studied the hippocampal tissue of suicide victims and found that *NR3C1* expression was decreased in only those suicide victims with a history of experiencing child abuse [32]. Palma-Gudiel et al. (2015) reviewed *NR3C1* methylation as a mediator of early adversity and found that early life stress was correlated with *NR3C1* hypermethylation at several CpG sites within the *NR3C1* 1F promoter region [63]. Radtke et al. (2015) found DNAm at the promoter region to be associated with child maltreatment and decreased psychological well-being [61]. Both Melas et al. 2013 and Tykra et al. (2012) reported that hypermethylation within the 1F promoter was associated with early parental loss, low levels of parental care, and childhood maltreatment [37,64,65]. These studies report changes in DNAm at CpG sites within the 1F promoter region. Our results differ in that cg08845721 (*NR3C1*) lies at the north shore of the CpG island that houses the 1F promoter region. However, the entire regulatory region, particularly the island shores, is also known to have *NR3C1* transcriptional importance.

DNAm at CpG islands is the area primarily thought to have a greater degree of influence on gene transcription and ultimately levels of protein production. However, recent research has shown the potential for greater functional importance of DNAm occurring at the proximal CpG island shore [66]. Importantly, Shields et al. 2016 examined the CpG island shore of *NR3C1* in 295 African American women and found that women reporting a history of childhood abuse had increased methylation at CpG site chr5:143401272 (Genome Reference Consortium Human Reference 38Genome: GRCh38) [67]. Using the UCSC Genome Browser’s Liftover Tool, our CpG site cg08845721 (chr5:142780693; GRCh37) converts to chr5:143401128 (GRCh38), only 144 bp downstream and within the regulatory proximal shore region, suggesting that DNAm at these and other CpG sites in the region may have functional importance. These intriguing findings require further study of CpG sites located within the island shore at the proximal promoter region of *NR3C1* to elucidate potential biomarkers that may identify survivors of ACEs at risk for prenatal depression.

Our finding of cg03238173 (*CRHR1*) as a mediator of the association of ACEs and depressive symptoms aligns with previous findings examining major depression. Researchers examining DNAm within *CRHR1* found associations with major depressive disorder (MDD). Interestingly, Humphreys et al. 2019 examined DNAm of *CRHR1* and found that the same CpG site we report (cg03238273) predicted MDD among adolescent girls [68]. Similarly, researchers examined of DNAm of *NR3C1* and *CRHR1* and found associations of DNAm of *NR3C1* and major depressive disorder (MDD) [69].

Understanding the neurobiological stress-response pathways linking ACEs to depressive symptoms may lead to a biomarker identifying women at risk of ACE-associated PD and support intensive monitoring strategies and early biopsychosocial interventions to decrease the risk of maternal depressive symptoms.

This study has several limitations, including its small sample size (*n* = 61), which limits generalizability and statistical power. Furthermore, the study was focused on specific genes to the exclusion of broader epigenetic or environmental factors. We did not control for current individual social circumstances (intimate partner violence, neighborhood characteristics, etc.) in the analysis due to limited statistical power in this exploratory study. Additionally, as the sample was composed predominantly of women with low household incomes, results may not be representative of the experiences of Black women more broadly. Future research should examine this association in light of current circumstances and in more diverse socioeconomic circumstances, as it is likely that there is a complex interplay between ACEs and adult life contexts due to the powerful contribution of ACEs to adverse behavioral and social determinants of health. ACEs measures fail to fully capture important sources of intersectional adversity, including experiences of racial discrimination, sex discrimination, and group-based or vicarious traumas such as systemic violence against Black people. Future research should examine the contributions of these additional sources of trauma toward mediating the relationship between ACEs and PD.

Despite these limitations, the findings of this study add to the current research focusing on the importance of the biological effect of environmental stressors on DNAm of glucocorticoid genes and the potential deleterious outcomes on mental health of Black pregnant women.

## 4. Materials and Methods

### 4.1. Design

Using a cross-sectional design, we examined depressive symptoms, ACEs, and DNAm in a subsample (*n* = 61) of Black pregnant women who were participants of the Biosocial Impacts of Black Births (BIBB) study. The BIBB study included women who were 8–22 weeks’ gestation at enrollment, 18–45 years of age, and who spoke and read English. Women who had multiple pregnancies (e.g., twins), fever at data collection, autoimmune disorders, or who received anti-inflammatory medications were excluded. Participants were recruited at prenatal clinics in Detroit, Michigan, and Columbus, Ohio. While data were collected at up to three time points, the ACEs scale was included in surveys only at the second time point. Therefore, the present study included a subsample of the 61 enrolled women who completed questionnaires at the second time point and had blood drawn for DNAm analysis. (Blood was only drawn for women recruited prior to the COVID-19 pandemic; enrollment 2018–March 2020.) Maternal medical and obstetric history were abstracted from medical records. The BIBB study obtained informed consent from all participants and was approved by the Institutional Review Boards of the participating universities and clinical sites.

### 4.2. Maternal Characteristics

Maternal sociodemographic characteristics were collected by self-report at the first time point and included maternal age, gestational age at data collection, marital status, employment status, level of education, and annual household income.

### 4.3. Adverse Childhood Experiences

The ACEs scale is a 10-item questionnaire that assesses childhood trauma [8,70] and was administered at the second time point. The questions assess experiences of childhood abuse and neglect as well as trauma experienced in the household prior to age 18 (e.g., Did a parent or adult in the household often or very often… Swear at you, insult you, put you down, or humiliate you? Did you live with anyone who was a problem drinker or alcoholic, or who used street drugs?). Participants answer yes = 1 or no = 0 for each item. Composite scores range from 0 to 10, with each affirmative answer indicating an occurrence of an ACE. Scores are reflected in a dose–response relationship with moderate (1–3) and high scores (≥4) associated with poorer health outcomes later in life [8,71,72]. The ACE scale had good internal consistency in this study (Cronbach’s α = 0.84).

### 4.4. Depressive Symptoms

The Center for Epidemiologic Studies Depression Scale (CES-D) is a 20-item, 4-point self-report questionnaire that measures the presence of levels of depressive symptoms within the past week [73]. Questions include, “I was bothered by things that usually, don’t bother me” “I felt depressed” and “I felt like everything I did was an effort.” Scores range from 0 to 60. Higher scores indicate a greater presence of depressive symptoms. A score of ≥16 indicates significant prenatal depressive symptoms, and a score of ≥23 is indicative of severe depressive symptoms [74]. The CES-D had good internal consistency in studies with Black pregnant women (Cronbach’s α = 0.89, 0.90) [75,76].

### 4.5. DNA Methylation

DNAm analysis was performed at Roswell Park Comprehensive Cancer Center’s Shared Genomics Core (Buffalo, NY, USA) using the Illumina Infinium EPIC 850K BeadChip array (Illumina, Inc., San Diego, CA, USA). A detailed description of DNAm processing is published elsewhere [77]. Average beta scores were calculated using Ge-nomeStudio Software v. 2.0, Illumina, Inc., San Diego, CA, USA) for each CpG site, and values assigned range from 0, indicating no methylation, to 1, indicating complete methylation. Beta scores for the candidate genes were utilized in the mediation analy-sis. Using the same model, we ran an exploratory epigenome-wide analysis of ACEs on DNAm to provide a global context. The data supporting this study have been deposit-ed in the NCBI Gene Expression Omnibus (GEO) under accession number [GSE311619].

### 4.6. Statistical Analysis

Maternal Characteristics. Descriptive statistics (e.g., mean, frequencies) were used to describe sample characteristics using SPSS, version 29. We tested the potential mediation of DNAm CpG sites in *FKBP5*, *NR3C1*, *CRH,* and *CRHR1* in the relationship between ACEs and CES-D using the “mediation” R package, v.4.5,1 [78].

Association between ACEs and depressive symptoms. Using a linear model, we tested the total effect of ACEs on depressive symptom scores (as a continuous variable). The model is $Y=\alpha_{0}+\alpha_{X}X+\epsilon$, where Y denotes the dependent variable, depressive symptoms score, and X denotes the independent variable ACEs, measured as a continuous variable ranging from 0 to 10. $\alpha_{0}$ and $\alpha_{X}$ denote the intercept and effect size of ACEs on depressive symptoms, and $\alpha_{X}$ is the total effect (TE) in the mediation analysis. We use a t-statistic from the regression to test the association coefficient $\alpha_{X}$ between ACEs and depressive symptoms and denote the corresponding *p*-value as $p_{\mathrm{TE}}$ for testing the TE $\alpha_{X}$.

Association between ACEs and DNAm. The following model was applied to test the association between ACEs and the DNAm CpG sites. $M=\beta_{0}+\beta_{M}X+\epsilon$, where M denotes the mediator DNAm; $\beta_{M}$ denotes the effect size of ACE on DNAm CpG.

Mediation effect of DNAm on ACEs associated with depressive symptoms. We simultaneously tested the effects of mediator (DNAm on the specific genes) and the independent variable (ACEs), both treated as continuous variables, on the dependent variable (depressive symptoms), also treated as continuous. We used the following model to obtain the mediation effect: $Y=\gamma_{0}+\gamma_{X}X+\gamma_{M}M+\epsilon$, where $\gamma_{X}$ and $\gamma_{M}$ denote the effect of ACEs and DNAm, respectively. The average direct effect (DE) and mediation effect (ME) are calculated by $\mathrm{DE}=\gamma_{X}$ and $\mathrm{ME}=\beta_{M}\times \gamma_{M}$, respectively. Samples with missing values for either DNAm or ACE group were excluded from each comparison. We used an alpha level of 0.05 to define statistical significance for these group comparisons.

## 5. Conclusions

Understanding the molecular pathways underlying the mediation of the effect of ACEs on depressive symptoms among Black pregnant women can illuminate biological markers that help identify and treat pregnant women who are at an increased risk for depression following childhood trauma. Identification of biomarkers will help to illuminate potential interventions—pharmacological, behavioral, or environmental support—to decrease risk prior to the onset of prenatal depression. Public health strategies informed by robust mechanistic approaches can better target screening, resilience-building programs (e.g., coping skills), ACEs prevention, and interventions to reduce the biological impact on the molecular pathways contributing to prenatal depression.

## Figures and Tables

**Table 1:** Maternal characteristics (*n* = 61).

Variable	N	Mean (SD) (Range)	Scores	Number (%)
**Maternal Age (years)**	61	27.8 (5.5) (18–41)		
**GA at Data**				
**Collection (weeks)**	60	13.6 (3.3) (8.3–22.2)		
ACEs *	49	2.4 (2.6)	0	8 (16.3)
		(0–4)	1–3	30 (61.2)
			4+	11 (22.5)
**CES-D ^+^**	59	17.2 (12.0)	<16	33 (55.9)
		(0–60)	≥16	26 (44.1)
			<23	42 (71.2)
			≥23	17 (28.8)
**Marital Status**	59			20 (34.0)
Married or Living w/Partner				20 (34.0)
Divorced/Separated				4 (6.7)
Never Married				35 (59.3)
**Employment**	61			
Working				32 (52.4)
Temporarily Laid-Off				4 (6.6)
Not Working				25 (41.0)
**Annual Household Income**	61			
<$10,000				21 (34.4)
$10,000–$30,000				31 (50.8)
$30,000–$39,999				3 (4.9)
$40,000–$59,999				4 (6.6)
>$60,000				2 (3.3)

* Adverse Childhood Experiences: Higher scores may indicate high risk of ACE-related health concerns. ^+^ Center for Epidemiologic Studies Depression Scale: A score of ≥16 indicates risk for depression. A score of ≤23 indicates risk for major depression.

**Table 2:** Mediation analysis of the effect of ACEs on depressive symptoms via DNA methylation.

Gene	CpG ^a^	CpG Location	Chromosomal Position	Relation to CpG Island	Total Effect	ME	DE	P_ME_	P_DE_
*CRHR1*	cg03238273	5′UTR	Ch.17:43825672	Ukn	2.287	−0.27	2.56	<0.001	2 × 10^−16^
*NR3C1*	cg08845721	5′UTR	Ch.5:142780693	N. Shore	2.287	−0.25	2.54	0.02	2 × 10^−16^
*NR3C1*	cg16594263	Body	Ch.5:142768048	Ukn	2.287	−0.26	2.54	0.04	2 × 10^−16^
*NR3C1*	cg19820298	Body	Ch.5:142770782	Ukn	2.287	0.47	1.81	<0.001	0.04
*NR3C1*	cg23430507	5′UTR	Ch.5:142798375	Ukn	2.287	−0.22	2.51	0.04	2 × 10^−16^

^a^ Cytosine-phosphate-guanine dinucleotide; ME: Average mediation effect; DE: Average direct effects; P_ME_: *p*-value of mediation effect; P_DE_: *p*-value of direct effect.

## Data Availability

All raw and processed data generated in this study have been de-posited in the NCBI Gene Expression Omnibus (GEO) [accession number: GSE311619].
